# Association of QT dispersion with mortality and arrhythmic events—A meta‐analysis of observational studies

**DOI:** 10.1002/joa3.12253

**Published:** 2019-11-11

**Authors:** George Bazoukis, Cynthia Yeung, Ryan Wui Hang Ho, Dimitrios Varrias, Stamatis Papadatos, Sharen Lee, Ka Ho Christien Li, Antigoni Sakellaropoulou, Athanasios Saplaouras, Panagiotis Kitsoulis, Konstantinos Vlachos, Konstantinos Lampropoulos, Costas Thomopoulos, Konstantinos P. Letsas, Tong Liu, Gary Tse

**Affiliations:** ^1^ Second Department of Cardiology Laboratory of Cardiac Electrophysiology "Evangelismos" General Hospital of Athens Athens Greece; ^2^ Department of Medicine Queen's University Kingston ON Canada; ^3^ Li Ka Shing Faculty of Medicine University of Hong Kong Hong Kong P.R. China; ^4^ Harvard Medical School Boston MA USA; ^5^ 3rd Department of Internal Medicine Sotiria General Hospital National and Kapodistrian University of Athens Medical School Athens Greece; ^6^ Laboratory of Cardiovascular Physiology Li Ka Shing Institute of Health Sciences Hong Kong S.A.R. P.R. China; ^7^ Faculty of Medicine Newcastle University Newcastle UK; ^8^ Laboratory of Anatomy‐Histology‐Embryology School of Medicine University of Ioannina Ioannina Greece; ^9^ Department of Cardiology Helena Venizelou Hospital Athens Greece; ^10^ Tianjin Key Laboratory of Ionic‐Molecular Function of Cardiovascular disease Department of Cardiology Tianjin Institute of Cardiology Second Hospital of Tianjin Medical University Tianjin China

**Keywords:** all‐cause mortality, arrhythmic events, coronary artery disease, heart failure, QT dispersion

## Abstract

**Background:**

The risk stratification of coronary heart disease (CHD) and/or heart failure (HF) patients with easily measured electrocardiographic markers is of clinical importance. The aim of this meta‐analysis is to indicate whether increased QT dispersion (QTd) is associated with fatal and nonfatal outcomes in patients with CHD and/or HF.

**Methods:**

We systematically searched MEDLINE and Cochrane databases without restrictions until August 15, 2018 using the keyword “QT dispersion”. Studies including data on the association between QTd and all‐cause mortality, sudden cardiac death (SCD) or arrhythmic events in patients with HF and/or CHD were classified as eligible.

**Results:**

In the analysis including patients with CHD and/or HF, we found that QTd did not differ significantly in patients with SCD compared to no SCD patients while QTd was significantly greater in the group of all‐cause mortality patients and in patients who experienced a sustained ventricular arrhythmia. Subgroup analysis showed that in myocardial infarction studies, QTd was significantly higher in patients with an arrhythmic event compared to arrhythmic event‐free patients while a nonsignificant difference was found in QTd in patients who died from any cause compared to survivors. Similarly, in HF patients, the QTd was significantly greater in patients with an arrhythmic event while a nonsignificant difference was found regarding all‐cause mortality and SCD outcomes.

**Conclusions:**

QTd has a prognostic role for stratifying myocardial infarction or HF patients who are at higher risk of arrhythmic events. However, no prognostic role was found regarding all‐cause mortality or SCD in this patient population.

## Graphical abstract

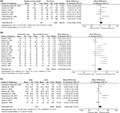

## 1 INTRODUCTION

The identification of patients at increased risk for sudden cardiac death (SCD) and major arrhythmic events [ventricular fibrillation (VF) and ventricular tachycardia (VT)] is of outstanding clinical importance. Noninvasive tests (signal‐averaged electrocardiography, arrhythmic burden in Holter monitoring, echocardiography markers) have been used to identify patients at high risk for fatal cardiovascular events.^1^, ^2^, ^3^ QT dispersion (QTd) (difference between maximum and minimum QT interval) is an easy measured electrocardiographic marker which correlates significantly with the dispersion of action potential duration at 90% repolarization and recovery time.^4^ Several studies have studied the association of QTd with arrhythmic events and mortality in different clinical settings,^5^, ^6^, ^7^, ^8^, ^9^, ^10^ including healthy men,^11^ elderly,^12^ and general population.^13^ However, the prognostic role of QTd is not yet fully established 0.^14^, ^15^ The risk stratification of coronary heart disease (CHD) or heart failure (HF) patients using easily measured electrocardiographic markers, such as QTd, would undoubtedly be a useful addition to the modern diagnostic arsenal. In order to aggregate diverging evidence in the field, we performed a quantitative synthesis of the existing data about the impact of QTd on three major outcomes (all‐cause mortality, SCD, and arrhythmic events) in patients CHD and/or HF.

## 2 MATERIALS AND METHODS

### 2.1 Search strategy

We systematically searched (WHHR, GB) MEDLINE (by using PubMed Web‐based search engine) and Cochrane databases without year, language, starting date, or any other restriction until August 15, 2018. We used the keyword “QT dispersion”. Furthermore, the reference lists of all included studies and relevant review studies were also searched to trace more eligible articles.

### 2.2 Study selection

The studies included in our meta‐analysis presented data concerning the association between QTd and all‐cause mortality, SCD or arrhythmic events in patients with HF or with CHD. For the classification of HF patients two criteria were used: mean EF <50% and type of cardiomyopathy (the presence of ischemic cardiomyopathy, dilated cardiomyopathy, or valvular heart disease in more than 85% of the total population included).

Studies were excluded from our analysis, using the following rejection criteria: (a) patients <18 years old, (b) no HF or CHD patients (according to the criteria we mentioned above), (c) hypertrophic cardiomyopathy or channelopathies in >15% of the included patients, (d) congenital heart disease, and (e) studies not providing full text in English language.

### 2.3 Data extraction

The data extraction performed by two independent investigators (GB and WHHR). The information extracted for each study included: (a) publication details (first author's last name, journal, year of publication), (b) general characteristics of the study (study design, follow‐up duration, number of patients), (c) characteristics of the study population [age, gender, type of cardiomyopathy, LVEF], and (d) QTd values in patients with all‐cause mortality, SCD, or arrhythmic events according to the definitions mentioned above.

### 2.4 Definitions

QTd was defined as the difference between the longest (QTmax) and the shortest (QTmin) QT intervals within a 12‐lead ECG.^16^ As arrhythmic events were considered VT or VF episodes. SCD was defined as an unexpected death because of cardiac causes (probably VT/VF or cardiac asystole leading to electromechanical dissociation) occurring in a short time period (generally within 1 h of symptom onset).

### 2.5 Statistical analysis

Data were analyzed using Review Manager software (RevMan, version 5.3; Oxford, UK). Continuous variables were pooled as mean differences. The statistical heterogeneity of the study was assessed using the I^2^ index. We considered low, medium, and high heterogeneity to have approximate values: 25% (I^2^ = 25), 50% (I^2^ = 50), and 75% (I^2^ = 75), respectively.^17^ Funnel plots were constructed to assess publication bias. Random effect models were utilized in the analysis because they provide a more conservative estimate of the overall results. Subgroup analysis regarding the underlying cardiac disease was a priori projected (MI studies, HF studies).

## 3 RESULTS

### 3.1 Search results

**Figure 1:**
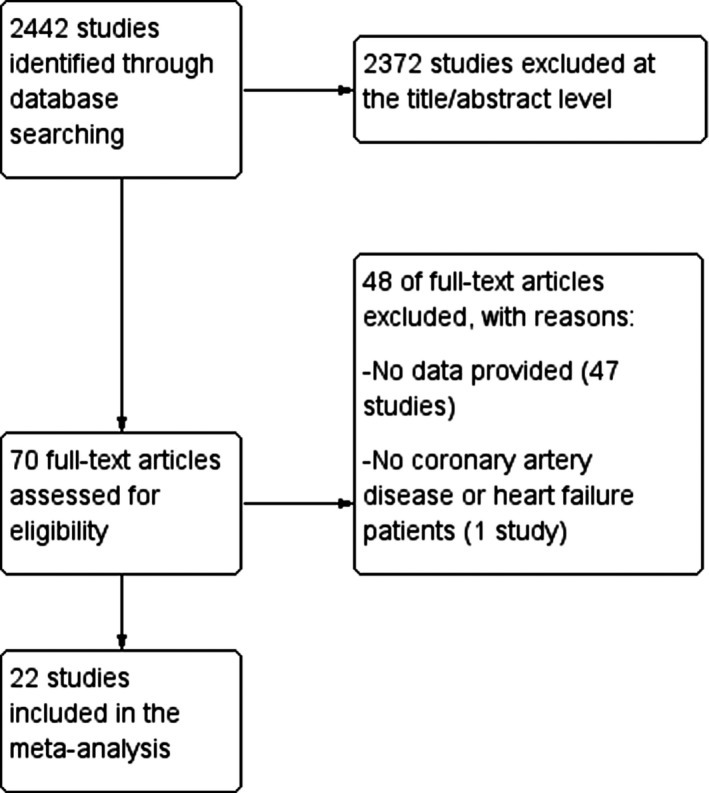
Flowchart of the search strategy

Our search strategy returned 2442 potentially relevant items (Figure 1). Of these, 22 studies^6^, ^7^, ^9^, ^18^, ^19^, ^20^, ^21^, ^22^, ^23^, ^24^, ^25^, ^26^, ^27^, ^28^, ^29^, ^30^, ^31^, ^32^, ^33^, ^34^, ^35^, ^36^ (5538 patients, mean age: 60 years old, 76.1% males) were finally included for further analysis. Seventeen studies included HF patients (3262 patients with ischemic heart disease, 862 patients with nonischemic heart disease, mean age: 62.8 years old, males: 76.3%, mean LVEF: 38.4%) according to the predefined criteria while 13 studies included only CHD patients. The baseline characteristics and the reported type of outcome of each included study are presented in Table 1.

**Table 1:** Baseline characteristics and reported outcomes of the included studies

First author/year	Enrollment period	Design	Single/multicenter	N	Males	Age (years)	Population included	Ischemic CMP	Nonischemic CMP	LVEF (%)	Follow‐up (months)	Deaths	Arrhythmic events (VT/VF)	Outcome reported
Mugnai G, 2016	January 2010 ‐ December 2012	Prospective	Single	223	171	64	MI (Anterior STEMI)	223	0	43	In hospital arrhythmia	14	33	Arrhythmic events
Zimarino M, 2011	April 2001‐ December 2003	Prospective	Single	612	508	63	PCI patients (primary/rescue/elective)	612	0	N/A	49	46 (25 SCD)	N/A	SCD
Tamaki S, 2009	N/A	Prospective	Single	106	81	64	HFrEF	55	51	30	65	38 (30 cardiac, 18 SCD)	N/A	SCD
Fauchier L, 2005	February 1987 ‐October 2001	Prospective	Single	162	138	51	Idiopathic DCM	0	162	33	53	30 (26 cardiac, 14 SCD)	9	Arrhythmic events
Huikuri HV, 2003	1996‐	Prospective	Single	650	489	61,8	MI	650	0	45,1	43	101 (59 cardiac, 22 SCD)	17	Arrhythmic events/ SCD
Gang Y, 2003 ELITE II	June 1997 ‐ May 1998	Prospective	Multi‐	986	703	71,2	HFrEF	769	217	31,3	17.8	140 (119 cardiac)	N/A	All‐cause mortality/ SCD
Kondo N, 2001	May 1997 ‐ January 2001	Prospective	Single	67	63	57	HFrEF	30	37	31	N/A	N/A	24	Arrhythmic events
Tapanainen JM, 2001	1996‐	Prospective	Single	379	272	62,4	MI	379	0	45	14	26 (18 cardiac)	N/A	All ‐cause mortality
Adachi K, 2001	February 1997 ‐ April 2000	Prospective	Single	64	52	49,1	DCM	0	64	45	24	1 SCD	9	Arrhythmic events
Spargias KS, 1999 AIRE, AIREX	N/A	Prospective	Multi‐	501	379	64,6	MI and HF	501	0	44,5	72	181	N/A	All‐cause mortality
Galinier M, 1998	January 1990‐ December 1995	Prospective	Single	205	170	58,2	HF (LVEF < 45%)	86	119	28,3	24	66 (56 cardiac)	7	All‐cause mortality, SCD, arrhythmic events
Trusz‐Gluza M, 1996	1986‐1990	Retrospective	Single	162	122	52,8	CAD	N/A	N/A	58,7	25	17, all cardiac (9 SCD)	46	All‐cause mortality, SCD
Perkiomaki JS, 1995	N/A	Retrospective Case‐control	Single	70	66	60,3	MI	70	0	45,3	N/A	N/A	30	Arrhythmic events
Anastasiou‐Nana MI, 2000	June 1993 ‐ July 1997	Prospective	Single	104	87	52,6	HFrEF	45	59	22	20	23, all cardiac (10 SCD)	N/A	All‐cause mortality, SCD
Grimm W, 1996	December 1992‐August 1995	Prospective	Single	107	81	48,8	Idiopathic DCM	0	107	31,2	13	5 SCD	7	Arrhythmic events
Yunus A, 1996	N/A	Retrospective Case‐control	Single	38	N/A	N/A	MI (STEMI)	38	0	N/A	N/A	N/A	19	Arrhythmic events
Glancy JM, 1995 LIMIT‐2	September, 1987‐ February, 1992	Retrospective	Single	326	220	67,4	MI	326	0	N/A	N/A	163	N/A	All‐cause mortality
Fiol M, 1995	N/A	N/A	N/A	246	N/A	N/A	MI	246	0	N/A	N/A	N/A	79	Arrhythmic events
Pye M, 1994^a^	N/A	Retrospective Case‐control	Single	89	65	62.9	MI and DCM	70	19	38,4	N/A	N/A	49	Arrhythmic events
Higham PD, 1995	N/A	Prospective	Single	30	N/A	N/A	MI	30	0	N/A	N/A	N/A	4	Arrhythmic events
Zabel M, 1998	1992 ‐ 1996	Prospective	Single	280	229	58	MI	280	0	47	32	21 (10 SCD and 6 pump failure)	9	All‐cause mortality, arrhythmic events
Fu GS, 1997	January 1990 – November 1992	Retrospective	Single	131	106	59,5	HFrEF	104	27	31	31,6	53 (49 cardiac)	10	Arrhythmic events

Abbreviations: CAD, coronary artery disease; CMP, cardiomyopathy; DCM, dilated cardiomyopathy; HFrEF, heart failure with reduced ejection fraction; LVEF, left ventricular ejection fraction; MI, myocardial infarction; PCI, percutaneous coronary intervention; SCD, sudden cardiac death; STEMI, ST‐elevation myocardial infarction; VF, ventricular fibrillation; VT, ventricular tachycardia.

a This study was used in two separate analyses regarding the type of cardiomyopathy.

### 3.2 Quantitative Synthesis

#### 3.2.1 Patients with coronary heart disease and/or heart failure

**Figure 2:**
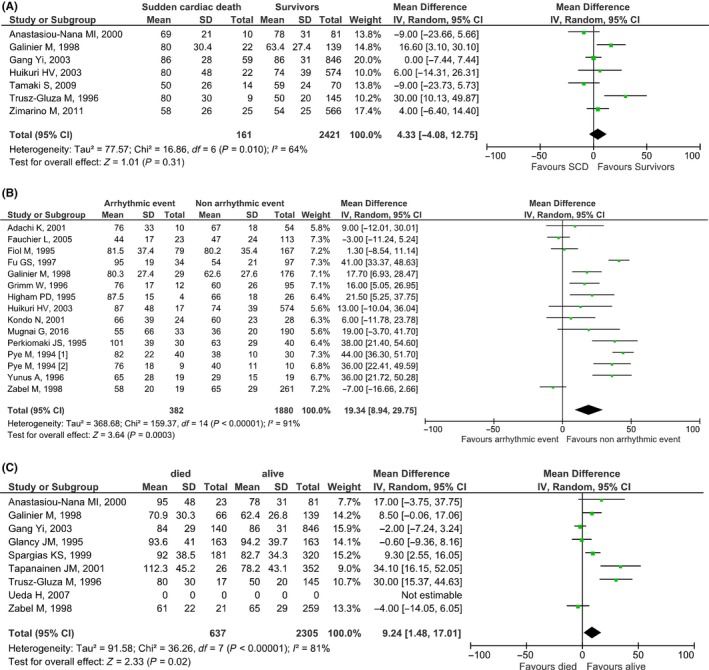
Forest plots regarding the association of QTd with (A) Sudden cardiac death, (B) arrhythmic events, (C) all‐cause mortality in coronary heart disease or/and heart failure patients

Our search retrieved seven studies (n = 2582 patients, age: 64.3 years old, males: 76.3%) including data regarding the association between QTd and SCD. No statistically significant difference was observed between patients with and without SCD (mean difference [95% CI]: 4.33 [−4.08, 12.75], *P* = .31, I^2^: 64%) (Figure 2A). Funnel plot showed no significant publication bias.

We found 14 studies (n = 2362 patients, mean age: 58.9 years old, males: 79.6%) including data regarding the association between QTd and arrhythmic events. The analysis showed that patients who at some point suffered from an arrhythmic event, have a significantly higher QTd compared to patients without arrhythmic events (mean difference [95% CI]: 19.34 [8.94, 29.75], *P* = .0003, I^2^: 91%) (Figure 2B). Funnel plot showed no significant publication bias.

Our search returned eight studies (n = 2943 patients, mean age: 64.7, males: 74.1%) including data regarding the association between QTd and all‐cause mortality. On these studies, survivors showed a marginal statistically significant difference in QTd when compared to all‐cause mortality patients (mean difference [95% CI]: 9.24 [1.48, 17.01], *P* = .02, I^2^: 81%) (Figure 2C). Funnel plot showed no significant publication bias.

#### 3.2.2 Subgroup analysis

In an attempt to review the literature in depth, we proceeded in a subgroup analysis, essentially separating the study population, previously described, in two major subgroups: Myocardial infarction (MI) patients and HF patients.

#### 3.2.3 Myocardial infarction patients

**Figure 3:**
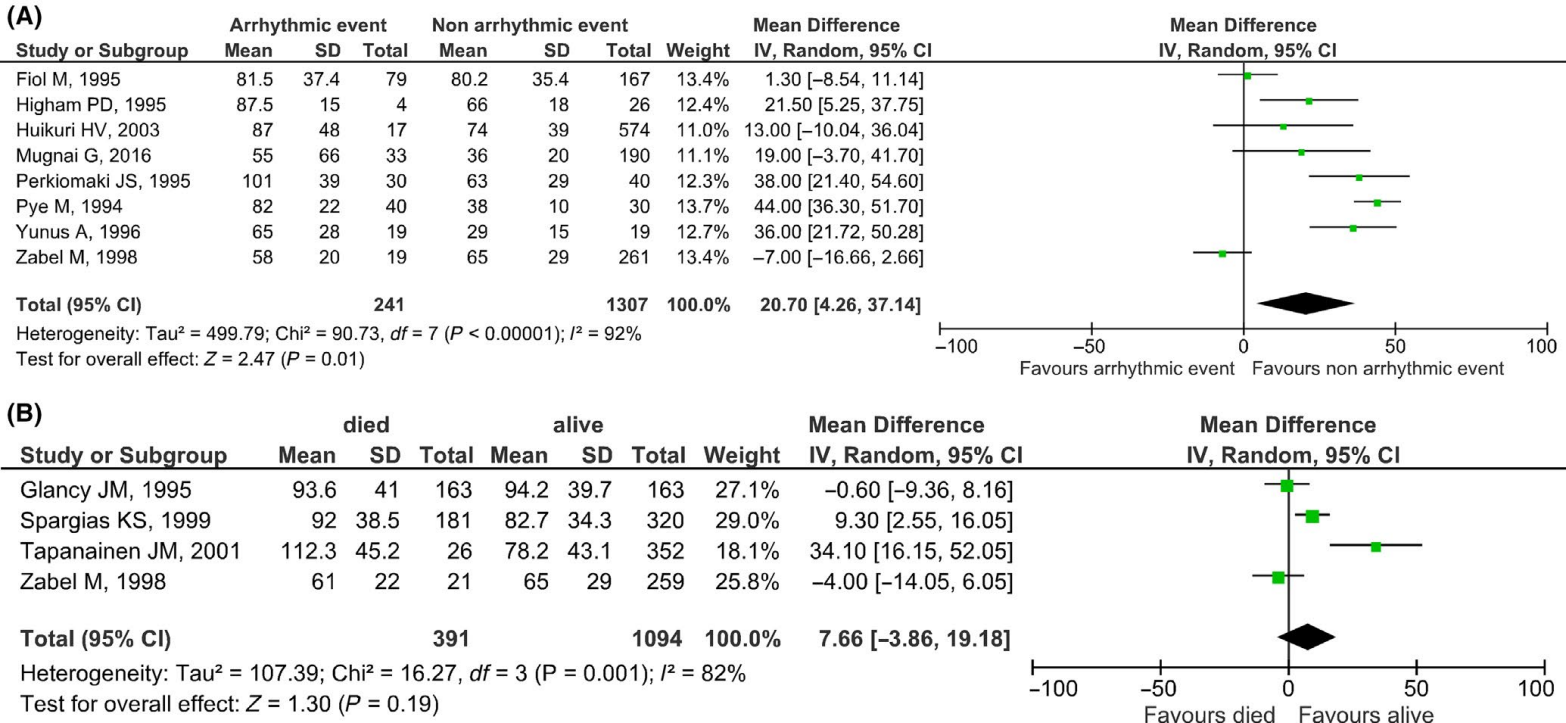
Forest plots regarding the association of QTd with (A) arrhythmic events, (B) all‐cause mortality in myocardial infarction patients

Our search retrieved eight studies (n = 1607 patients, mean age: 65.6 years old, males: 65.6%) including data concerning the association between QTd and arrhythmic events in MI patients. MI patients with arrhythmic events had significantly higher QTd when compared to MI patients without major arrhythmic events (mean difference [95% CI]: 20.70 [4.26, 37.14], *P* = .01, I^2^: 92%) (Figure 3A). On the other hand, the quantitative synthesis of the four studies (n = 1486, mean age: 63.4 years old, males: 74%) including data about QTd and all‐cause mortality showed no significant difference (mean difference [95% CI]: 7.66 [−3.86, 19.18], *P* = .19, I^2^: 82%) (Figure 3B). Funnel plots showed no significant publication bias. Our search for studies associating QTd with SCD in MI patients yielded only one result, therefore, that is not presented in our analysis.

#### 3.2.4 Heart Failure Patients

**Figure 4:**
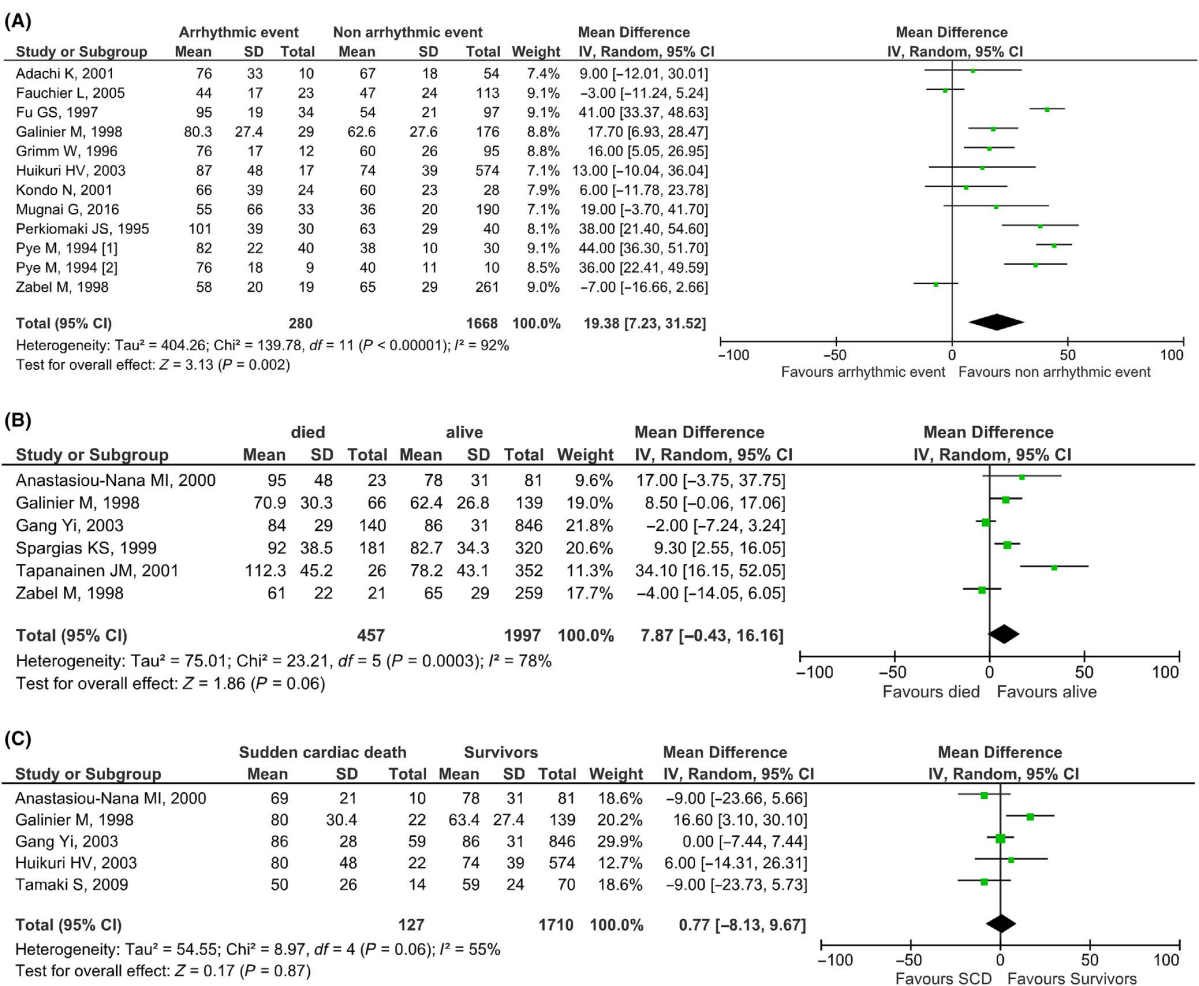
Forest plots regarding the association of QTd with (A) arrhythmic events, (B) all‐cause mortality, (c) sudden cardiac death in heart failure patients

Our search retrieved 11 studies (n = 2048 patients, mean age: 58.9 years old, males: 79.6%, ischemic cardiomyopathy: 73.9%, mean LVEF: 40.3%) including data about the association of QTd with arrhythmic events in HF patients. We found a statistically significant difference in QTd between HF patients with an arrhythmic event and those patients who were arrhythmia free (mean difference [95% CI]: 19.38 [7.23, 31.52], *P* = .002, I^2^: 92%) (Figure 4A).

Six studies (n = 2455 patients, mean age: 65.1 years old, males: 74.8%, ischemic cardiomyopathy: 83.9%, mean LVEF: 37.3%) reported information on all‐cause mortality but there was no statistically significant difference in QTd when associating QTd with all‐cause mortality outcome (mean difference [95% CI]: 7.87 [−0.43, 16.16], *P* = .06, I^2^: 78%) (Figure 4B). Five studies reported SCD outcomes (n = 2051 patients, mean age: 65.6 years old, males: 74.4%, ischemic cardiomyopathy: 78.3%, mean LVEF: 34%) but our meta‐analysis similarly did not find a difference in QTd between patients who died of SCD and those who did not (mean difference [95% CI]: 0.77 [−8.13, 9.67], *P* = .87, I^2^: 55%) (Figure 4C).

### 3.3 Quality assessment

The Newcastle‐Ottawa Quality Assessment Scale (NOS) was used for quality assessment of the included studies.^37^ The NOS point score system evaluated the categories of study participant selection, comparability of the results, and quality of the outcomes. The following characteristics were assessed: (a) representativeness of the exposed cohort; (b) selection of the nonexposed cohort; (c) ascertainment of exposure; (d) demonstration that outcome of interest was not present at the start of study; (e) comparability of cohorts based on study design or analysis; (f) assessment of outcomes; g) follow‐up periods that were sufficiently long for outcomes to occur; and (h) adequacy of follow‐up of cohorts. This scale ranged from zero to nine stars, which indicated that studies were graded as poor quality if the score was <5, fair if the score was 5 to 7, and good if the score was >8. All included studies were graded with a score >5 while none of the included studies was characterized as having a poor quality (Table S1).

## 4 DISCUSSION

### 4.1 Rational

Existing literature hinds toward the role of QT dispersion in predicting the trajectory of cardiovascular disease. Yet, a definitive model that would shape current guidelines and would help clinicians with stratification of risk for patients with HF and CAD remains a challenge.

Analyzing the 22 included studies we concluded to the following (a) QTd was significantly associated with arrhythmic events and all‐cause mortality but not with SCD in CHD and/or HF patients (b) In the MI group, we found that higher QTd was associated with increased incidence of arrhythmic events but not associated with increase in all‐cause mortality. iii) In the HF group, we found a significant association between QTd and arrhythmic events but we failed to prove an association between QTd and SCD or all‐cause mortality. An explanation about the significant association of QTd with arrhythmic events and not with SCD in CHD and/or HF patients is the large number of arrhythmic events compared to the small number of SCDs. This means that fewer studies are needed to reach in a statistically significant result about the association of QTd with arrhythmic events compared with SCD. Another explanation is that most of the studies which provided data about arrhythmic events did not provide data for SCD and vice versa.

### 4.2 Pathophysiologic mechanism

Identification of the exact role of QTd in arrhythmogenesis remains a challenge. QTd contributes the heterogeneities of repolarization time in the three‐dimensional structure of the ventricular myocardium, which are secondary to regional differences in action potential duration and activation time.^38^ The increased QTd seems to act in the absence of abnormalities that have been proposed by Packer et al as mechanisms of sudden death in patients with congestive HF (abnormality in neurohormones, electrolytes, or wall stress).^39^, ^40^ An explanation about the association between QTd and arrhythmogenesis is the increased disparity of regional ventricular repolarization times which predisposes to sustained ventricular arrhythmias.^41^ The causative role of this inhomogeneity on ventricular arrhythmia has been shown in experimental studies and during programmed electrophysiological studies in humans.^42^, ^43^

QTc dispersion has been found to be prolonged in acute MI while the QTc dispersion influenced by the site and size of infarction.^44^, ^45^ Myocardial fibrosis could be one local factor leading to greater QTd in these patients.^40^ It has been found that in the acute setting of MI, the QTd gradually increases, peaks at day 3 and then falls after few days in most cases.^46^, ^47^ As a result, the timing of QT measurements has great importance after MI. Another interesting finding is that the beneficial role of classic HF medications may be attributed to the effect of these drugs in QTd shortening.^48^ For example, the beneficial role of carvedilol in HF patients has been attributed to the dose‐dependent reduction of QTd.^48^ The beneficial effect of drug combinations in QTd in HF patients has also been studied.^49^

### 4.3 Associations

QTd has been associated with arrhythmic events in long QT syndrome, HF patients, CHD, post‐MI or hypertrophic cardiomyopathy.^14^ A positive association between QTd and left ventricular ejection fraction has been demonstrated in MI patients.^50^ Additionally, the computerized measurements of QTd have been proposed as a tool for noninvasive risk stratification of patients at higher risk of cardiovascular mortality as indicated by the results of the community‐based Strong Heart Study,^51^ while the prolongation of the corrected QTd after hemodialysis has been found to predict cardiovascular mortality in hemodialysis patients.^52^ Other repolarization markers such as T_peak_‐T_end_ interval have been found to be significant higher in individuals who are at elevated risk for adverse events in congenital LQTS.^53^ In the same context, late potentials have been associated with ventricular arrhythmia in patients with MI but there was no correlation between late potentials and QTd, possibly because of the fact that they reflect different electrophysiological disorders (late potentials identify mainly depolarization disorders while QT dispersion is related to repolarization disorders).^45^

Several studies reported a significant shortening in QTd following successful coronary revascularization or thrombolysis.^47^, ^54^ The QTd shortening and the subsequent reduction of ventricular arrhythmia inducibility may be one of the mechanisms of arrhythmic events reduction in patients undergo thrombolytic or percutaneous coronary intervention (PCI) treatment. In particular, the absolute corrected QTd change after PCI has been found to be significantly correlated with major adverse cardiac events in patients with ST‐elevation MI.^55^ Another study concluded that early QTd reduction after primary PCI has been found to be closely related to the restoration of reperfusion at the microvascular level and provides additional prognostic information in ST‐elevation MI patients.^56^ Furthermore, a significant decrease in QTd has been proposed to provide an additional electrocardiographic index for successful (TIMI 2/3) reperfusion.^57^

Regarding HF patients, there are two large prospective studies measured the association between QTd and outcomes in HF patients. In the Danish Investigations of Arrhythmia and Mortality on Dofetilide congestive heart failure (Diamond‐CHF) substudy, QTd had no prognostic role on all‐cause mortality, cardiac mortality, or cardiac arrhythmic mortality in 703 patients with advanced congestive HF.^15^ In the United Kingdom Heart Failure Evaluation and Assessment of Risk Trial (UK‐HEART) study, the QT parameters failed to independently predict all‐cause mortality, SCD, or progressive HF death in 495 patients with mild or moderate HF.^58^ Both of these studies agree with the results of our meta‐analysis concerning the association of QTd with all‐cause mortality. There are different potential reasons as to why this is the case. To begin with, clinic‐based studies are potentially more biased towards having the greater proportion of patients with more comorbidities. Secondly, different cut‐off values can alter the hazard ratios. Finally, intraindividual variations in QTd means that QTd can be over‐ or under‐estimated.

Interestingly, the prognostic significance of QTd in HF patients seems to be related with the type of HF.^23^ Specifically, Galinier M et al, showed that QTd had a prognostic role in regard to arrhythmic events in dilated cardiomyopathy patients while no significant association was found in ischemic cardiomyopathy patients.^23^

## 5 CONCLUSIONS

QTd is associated with a higher incidence of major arrhythmic events in patients with HF or MI but is not associated with an increased incidence of SCD or all‐cause mortality in this population.

## 6 LIMITATIONS

Our study included observational studies some of them with retrospective analysis of their data which may introduce an element of bias. However, by excluding the retrospective studies, we retrieved similar results. Potential unmeasured external confounding factors could pose another limitation to our analysis. In addition, our study assumed that all QTd measurements in between studies where performed with the same accuracy, which might differ in reality. Digitalized measurement of the repolarization variables failed to show a prognostic role of QTd in post‐MI patients.^35^ Furthermore, the timing of QT measurement after MI is a parameter to consider, as a time‐dependent change in QT has been found after MI.^46^, ^47^ On the other hand, the circadian variation of QTd has been found in healthy subjects and in patients with uncomplicated CHD, but not in those who had suffered a previous MI and in patients with HF.^59^ As a result, we believe that the timing of QT measurements in our patient population does not pose a significant bias in our study. Another possible limitation is the presence of bundle branch block or not. However, studies have shown that QTd seems to not be influenced by the presence of bundle branch block.^58^ Moreover, another possible confounder that may influence our results, is the impact of cardiologic and noncardiological medications and the different clinical conditions (autonomic dysfunction, hemodialysis, electrolyte disturbances etc) in QTd.^60^, ^61^, ^62^, ^63^, ^64^ Regarding the arrhythmic event outcome, we defined as arrhythmic event, the occurrence of VT or VF. However, we included three studies^9^, ^22^, ^35^ that defined arrhythmic event as VT/VF or SCD and included in total 39 SCDs which consisted 10.2% of the total arrhythmic event patients. However, by excluding these studies, we received similar results (mean difference [95% CI]: 20.03 [8.64, 31.43], *P* < .001, I^2^: 89%). Additionally, our search did not retrieve enough data about the association of corrected QTd with the major outcomes. As a result, we included only QTd data in the quantitative synthesis. We did not perform a subgroup analysis regarding the type of HF (ischemic vs nonischemic) because of the small number of studies. Two studies (Huikuri et al^26^ and Tapananien 32 et al) include overlapping cohorts but they were used in different outcomes analyses. Finally, although a number of patients in the Kondo N et al^27^ study had a nonsustained VT, they included in the arrhythmic event analysis.

## CONFLICTS OF INTEREST

None.
