# Supplementary material for: Association of QT dispersion with mortality and arrhythmic events—A meta‐analysis of observational studies
Source: J Arrhythm. 2019 Nov 11;36(1):105–15. doi: 10.1002/joa3.12253 (PMC7011802; doi:10.1002/joa3.12253)
Supplement: Supplementary file 1 [file JOA3-36-105-s001.docx]

| **Prospective studies** | | | | | | | | | | | | | | |
| --- | --- | --- | --- | --- | --- | --- | --- | --- | --- | --- | --- | --- | --- | --- |
|  | Selection | | | | | | | Comparability | Outcome | | | | |  |
| STUDY | Representativeness of the exposed cohort | Selection of the non-exposed cohort | | Ascertainment of exposure | | Demonstration that outcome of interest was not present at start of study | | Comparability of cohorts on the basis of the design or analysis | Assessment of outcome | Was follow-up long enough for outcomes to occur | | Adequacy of follow up of cohorts | | Total stars |
| Adachi K, 2001 | * | * | | * | | * | | ** | * | * | | * | | 9 |
| Anastasiou-Nana MI, 2000 | * | * | | * | | * | | ** | * | * | | * | | 9 |
| Galinier M, 1998 | * | * | | * | | * | | ** | * | * | | * | | 9 |
| Gang Yi, 2003 | * | * | | * | | * | | ** | * | * | | * | | 9 |
| Grimm W, 1996 | * | * | | * | | * | | ** | * | * | | * | | 9 |
| Higham PD, 1995 | * | * | | * | | * | |  | * | * | | * | | 7 |
| Huikuri HV, 2003 | * | * | | * | | * | | ** | * | * | | * | | 9 |
| Mugnai G, 2016 | * | * | | * | | * | | ** | * | * | | * | | 9 |
| Spargias KS, 1999 | * | * | | * | | * | | ** | * | * | | * | | 9 |
| Tamaki S, 2009 | * | * | | * | | * | | ** | * | * | | * | | 9 |
| Tapanainen JM, 2001 | * | * | | * | | * | | ** | * | * | | * | | 9 |
| Zabel M, 1998 | * | * | | * | | * | | ** | * | * | | * | | 9 |
| Zimarino M, 2011 | * | * | | * | | * | | ** | * | * | | * | | 9 |
| **Retrospective studies** | | | | | | | | | | | | | | |
|  | Selection | | | | | | | Comparability | Outcome | | | | |  |
| STUDY | Is the case definition adequate? | | Representativeness of the cases | | Selection of Controls | | Definition of Controls | Comparability of cases and controls on the basis of the design or analysis | Ascertainment of exposure | | Same method of ascertainment for cases and controls | | Non-Response rate | Total stars |
| Fauchier L, 2005 | * | | * | | * | | * | ** | * | | * | | * | 9 |
| Fiol M, 1995 | * | | * | | * | | * |  | * | | * | | * | 7 |
| Fu GS, 1997 | * | | * | | * | | * | ** | * | | * | | * | 9 |
| Glancy JM, 1995 | * | | * | | * | | * | ** | * | | * | | * | 9 |
| Kondo N, 2001 | * | | * | | * | | * | ** | * | | * | | * | 9 |
| Perkiomaki JS, 1995 | * | | * | |  | | * | * | * | | * | | * | 8 |
| Pye M, 1994 | * | | * | |  | | * |  | * | | * | | * | 6 |
| Trusz-Gluza M, 1996 | * | | * | | * | | * | ** | * | | * | | * | 9 |
| Yunus A, 1996 | * | | * | | * | | * | ** | * | | * | | * | 9 |
